# Control of the Singular Points Location for Miniature Switches with Magnetically Driven Contacts †

**DOI:** 10.3390/s18020350

**Published:** 2018-01-26

**Authors:** Xin Wang, Marcin Habrych, Bogdan Miedzinski, Julian Wosik

**Affiliations:** 1 Beijing Key Laboratory of Optimized Design for Modern Agricultural Equipment, College of Engineering, China Agricultural University (East Campus), 100083 Beijing, China; 2 Department of Electrical Power Engineering, Wroclaw University of Science and Technology, Wybrzeze Wyspianskiego 27, 50-370 Wroclaw, Poland; marcin.habrych@pwr.edu.pl; 3 Institute of Innovative Technologies EMAG, Leopolda 31, 40-189 Katowice, Poland; bogdan.miedzinski@pwr.edu.pl (B.M.); julianwosik@wp.pl (J.W.)

**Keywords:** miniature reed switch, MEMS actuator, magnetically driven contact, singular point location, dc magnetic field

## Abstract

This paper presents and discusses usefulness and possibility of control of the singular points location of a driving magnetic field under as structure considerations as well as selection of energizing magnet systems for miniature electromagnetic switches. The sample results of theoretical analysis and experimental testing concern selected reed switches with normally open contacts as well as a developed miniature switch with a metallic ball contact. It must be noted that the switch with the contact performed by means of the metallic tiny ball can be effectively used both as a change-over switch as well as a detector of the energizing magnetic field distribution under designing and assembling of any electromagnetic contact device preferably with an increased degree of integration and miniaturization.

## 1. Introduction

Miniaturization and integration of various devices and equipment is necessary for the development of any advanced technical structure to meet ever higher requirements. This applies, among others, to such elements as various miniature connectors, contact switches, (both so-called dry and wetted with mercury), valves (hydraulic, pneumatic) etc. Any manually and/or automatically controlled actuators, single or multi-position sensors as well as other types of electromagnetic relays are also products sought on the market. Do not forget about the reed contacts, which miniature products in 5–7 mm glass housings produced traditionally still enjoy success [1,2,3]. The micromechanical structures (so-called MEMS connectors) are also of great importance. Volume of their magnetic driving systems does not exceed 2–3 mm^3^ [4,5,6,7,8,9,10]. It should be emphasized that the use of micro-machine technology allowed for a significant miniaturization of the various device structures, for example the reed contacts of about 2 × 1.4 × 0.75 mm [11,12]. An interesting product is a miniature switch with a ferromagnetic ball of a diameter of 2–3 mm that serves as the magnetic system as well as the electrical contact [13]. The control of all above-mentioned devices is by means of a DC magnetic field produced by coils (also made by an electron beam technology [6,9,10,14]) and/or by the movable permanent magnets [1,2]. It should be emphasized however, that in the case of the permanent magnets, the increase of the sensitivity with simultaneous miniaturization requires the minimized length of the so-called “differential movement” of the control element (s) [3,15]. This is usually accomplished by the increase of the magnetic field gradient distributed along the direction of motion of the energizing element. This obviously requires reliable information on the magnetic field distribution with respect to the designed switch structure. It should be noted here, that the requirements for miniaturized devices (addressed for medical, automotive and other micro switching applications [6,10]) are quite opposite. On the one hand, it is required to significantly reduce the dimensions and on the other hand, the great reliability and sensitivity of operation, the appropriate ability and durability of switching and the electric strength of the contact gap. This requires, therefore, a sufficiently high value of the contact force and a significant speed of movement of the contacts during operation. This is quite difficult to obtain due to the small values of the length of movement of the driving magnet [2]. In connection with the above, it is aimed at providing significant values of both magnetic flux density and a significant gradient of its change within a small operational area. Therefore, the problem of theoretical determination and experimental confirmation of this type of areas becomes of great importance. These areas occur near the so-called singular points in which the magnetic field intensity tends to be zero. Unfortunately, this fact is not implemented sufficiently under designing.

In the article, an appropriate approach to the design of selected miniature structures taking into account distribution of the singular points (on the example of the control of a reed switch and a sensor with a ball contact) is presented. A mathematical method of this approach is discussed and verified by the measurements. As a result, the conclusions and recommendation for use in practice are formulated.

## 2. Mathematical Modelling of the Magnetic Field for the Selected Switch Structures 

**Figure 1:**
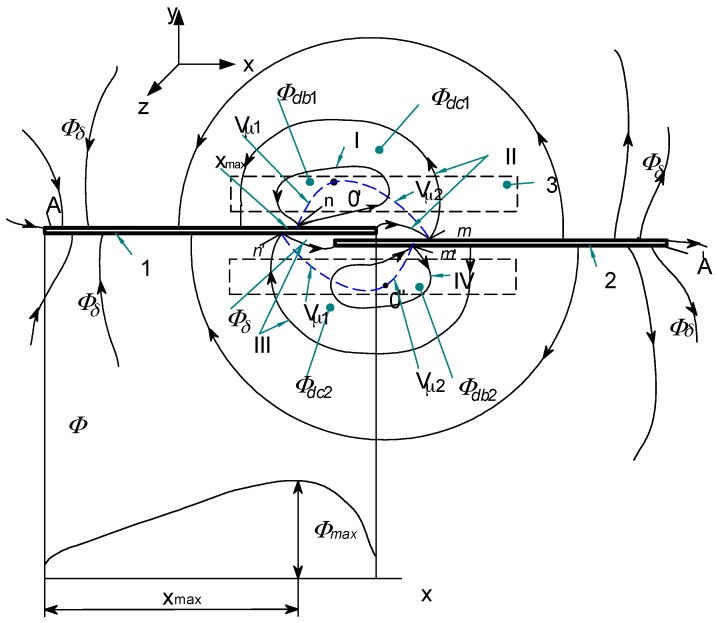
Distribution of the magnetic field (flux density lines) of the reed switch operated by the DC coil, on the *x-y* plane; 1,2—blades; 3—driving coil; I–IV separation lines—gradient line; (potential V), *n*, *n*’, *m*, *m’*—singular points.

Analysis of operation of any switch with magnetically controlled contacts depends on its structure as well as on operation principle. In any case, however, it is necessary to know the magnetic field distribution, which for the simple reed switch (with an open contact) when controlled by a DC coil is shown, for example, in Figure 1 [2].

**Figure 2:**
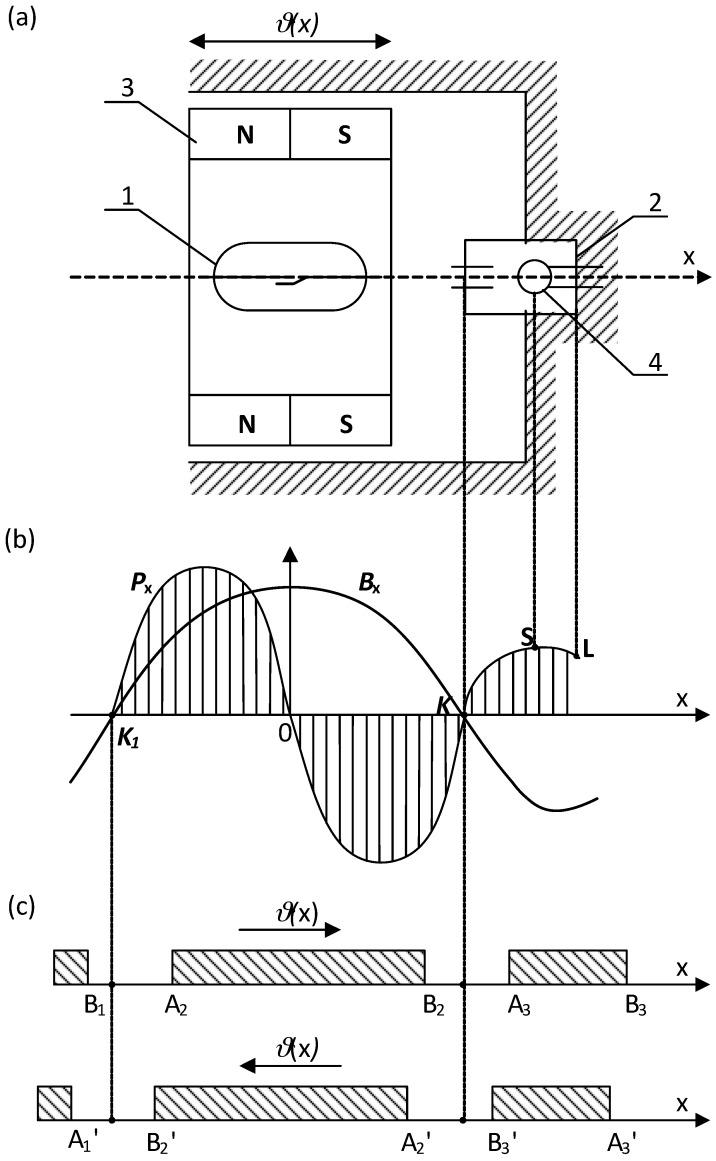
The reed (1) and/or the ball contact sensor (2) controlled by means of a movable (*ϑ*(*x*)) single ring magnet (3) magnetized axially (**a**); (**b**)—flux density *B_x_* distribution and a force *P_x_* affecting the ball (4); (**c**)—areas of the reed switch operation: A_1_…A_3_, B_1_…B_3_ contact closure and release respectively; *K*, *K*_1_-singular points.

**Figure 3:**
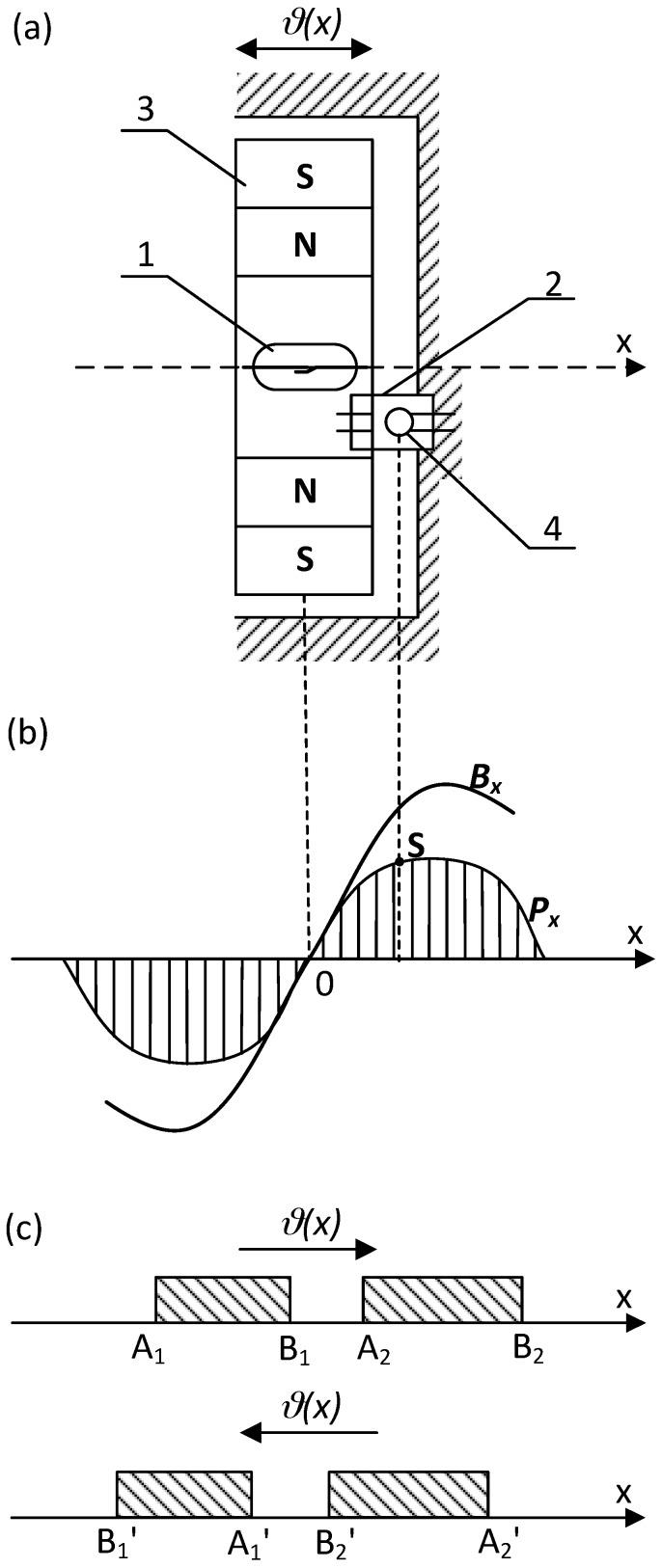
The reed (1) and/or the ball contact sensor (2) controlled by means of a movable (*ϑ*(*x*)) single ring magnet (3) magnetized vertically (**a**); (**b**)—flux density *B_x_* distribution and a force *P_x_* affecting the ball (4); (**c**)—areas of the reed switch operation: A_1_…A_2_, B_1_…B_2_ contact closure and release respectively; *K*-singular point.

From the presented picture of the magnetic field (for the symmetrical reed contact placed centrally inside the coil), it can be seen that the separation lines I–IV distribute the characteristic areas of the magnetic fluxes [2]. Inside the lines I and IV there are located leakage fluxes *Φ_db_*_1_ and *Φ_db_*_2_ that pass only through the air or the winding. However, between separation lines I and II there is an area of the leakage flux *Φ_dc_*_1_ that passes both through the air and the blade 1. A similar area of the leakage flux *Φ_dc_*_2_ that flows through the air and the blade 2 is located between the separation lines III and IV. The area for the effective flux *Φ_δ_*, which goes through both blades 1 and 2 and the contact gap *δ*, is restricted by the lines II and III respectively. From the analysis of the field picture one can estimate the magnetic flux distribution inside the blade (in its cross-section) along the *x*-axis. This is illustrated by the curve *Φ_x_*(*x*) shown at the lower part of Figure 1. As follows from the above, the control of as distribution as well as value of the magnetic field inside the contact gap area requires proper location of the reed switch inside the coil. In the case that any of the singular points *n*...*m* is located inside the center of the contact gap, the gap value will be kept constant and the rated due to lack of the residual flux. The longest gap value provides the highest electrical strength of the open reed switch. It should be noted here that the change in the blade’s polarity (due to the magnetizing effect) does not affect the performance of the reed switch due to the principle of its operation. However, for the devices that are controlled by a coil, the adjustment of the singular points is neither simple nor sometimes possible. The best suited devices are these with the movable permanent magnets. For example, Figure 2 and Figure 3 select the simple structures with movable permanent magnets (magnetized axially as well as vertically) driving a miniature reed switch and/or a miniature ball contact sensor for the analysis.

In the ball contact switch, the ball made of a soft ferromagnetic material meets a function of both the magnetic circuit and the electric change-over contact. Because of a low value of the contact force, the ball may be wetted additionally with mercury. For the reed switch, the force between the blade tips depends on the square of the effective flux value in the contact gap and changes respectively to variation of a magnetic conductivity of the resultant magnetic system with the distance between contacts under operation. It does not depend on the direction of the magnetic flux, hence on the polarity of the magnetized blades. Of course, the flux variation rate inside the contact gap affects the reed dynamics in transient [1,2,3]. Thus, the operation of the reed switch during the movement of the permanent magnet occurs after passing the singular points (*K*, *K*’) location irrespective of the flux direction. It is indicated schematically in Figure 2c and Figure 3c respectively. However, the situation is different for the ball micro-switch since the ball changes its position with respect to the flux direction. In order to simplify the analysis, the small geometric dimensions of the ball (not preliminary magnetized) has been assumed. So if the ball, made of a material with a high magnetic permeability, is uniformly magnetized and its geometrical dimensions are small with respect to the magnet, as well, thus the force *P_x_* affecting it (due to the external field) along the *x*-axis can be derived from following formula:

(1) $$P_{x}=\frac{4\pi }{\mu }R_{B}^{3}B\frac{\partial B}{\partial x}$$

where: *µ*—permeability of the ball material, *R_B_*—ball radius, *B*—value of the magnetic field density at the considered location.

From formula (1) it follows that the value of the force *P_x_* can be estimated for known distribution of both the magnetic flux density value and its rate change with the location of the driving magnet on the required *x*-axis. To determine the magnetic field distribution at the analyzed area, it is convenient to use a method of an equivalent solenoid. According to this theory, any permanent magnet of any shape (block, cylinder and/or ring) magnetized along the axis of symmetry can be replaced by a respective thin solenoid of the same size as the magnet [3,15,16]. It can also be modeled in the form of two inversely located thin ring solenoids where, the ampere-turns (MMF) value Θ of the equivalent solenoid and magnetization *M* of a uniformly magnetized magnet are related mutually as follows:

(2) Θ=M2c

where: 2*c* is the size of the magnet according to direction of magnetization.

Value of the elementary magnetic flux density *dB* due to the current *I* in the conducting filament *dl* at any point *P* (*x*_0_, *y*_0_, *z*_0_) can be calculated in accordance with the Biot-Savart-Laplace law:

(3) $$\bar{dB}=\frac{\mu }{4\pi }\frac{I}{r^{3}}\left[\bar{dl},\bar{r}\right]$$

where: *r*—radius as a vector directed from *dl* to *Р* point.

**Figure 4:**
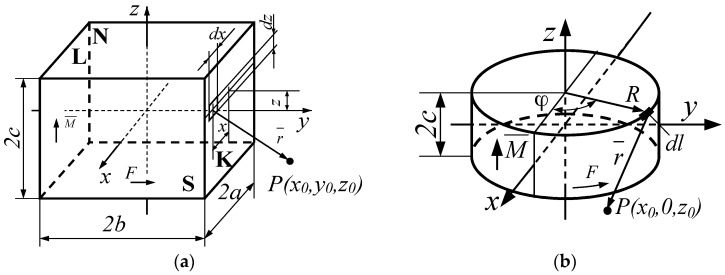
Illustration for calculation of the magnetic field contribution due to the permanent magnet; (**a**) rectangular; (**b**) cylindrical shape.

The resultant induction value is therefore found after integration of Equation (3) to estimate the contribution due to the total surface area of the equivalent solenoid. For example, a rectangular permanent magnet of side lengths equal to 2*a*, 2*b* and 2*c*, and magnetized along the *z*-axis, can be replaced by the solenoid of the same shape with respective currents flowing in the contour of sides **K**, **L**, **S** and **N**, respectively, as shown in Figure 4.

The expression for calculation of the flux density components *B_x_*_,*K*_ *B_y_*_,*K*_ and *B_z_*_,*K*_ along the *x*, *y* and *z*-axis due to the **K** surface at any point of a space is obtained after double integration over the **K** surface:

(4) $$B_{x,K}=0\;B_{y,K}=\frac{\mu F}{8\pi c}\ln\left[\begin{array}{l} \frac{\left|x_{0}-a+\sqrt{{\left(x_{0}-a\right)}^{2}+{\left(y_{0}-b\right)}^{2}+{\left(z_{0}-c\right)}^{2}}\right|}{\left|x_{0}+a+\sqrt{{\left(x_{0}+a\right)}^{2}+{\left(y_{0}-b\right)}^{2}+{\left(z_{0}-c\right)}^{2}}\right|}\\ \times \frac{\left|x_{0}+a+\sqrt{{\left(x_{0}+a\right)}^{2}+{\left(y_{0}-b\right)}^{2}+{\left(z_{0}+c\right)}^{2}}\right|}{\left|x_{0}-a+\sqrt{{\left(x_{0}-a\right)}^{2}+{\left(y_{0}-b\right)}^{2}+{\left(z_{0}+c\right)}^{2}}\right|} \end{array}\right]\;\begin{array}{c} B_{z,K}=\frac{\mu F}{8\pi c}[arctg\frac{\left(x_{0}-a)(z_{0}-c\right)}{(y_{0}-b)\sqrt{{\left(x_{0}-a\right)}^{2}+{\left(y_{0}-b\right)}^{2}+{\left(z_{0}-c\right)}^{2}}}\\ +arctg\frac{\left(x_{0}+a)(z_{0}+c\right)}{(y_{0}-b)\sqrt{{\left(x_{0}+a\right)}^{2}+{\left(y_{0}-b\right)}^{2}+{\left(z_{0}+c\right)}^{2}}}\\ -arctg\frac{\left(x_{0}-a)(z_{0}+c\right)}{(y_{0}-b)\sqrt{{\left(x_{0}-a\right)}^{2}+{\left(y_{0}-b\right)}^{2}+{\left(z_{0}+c\right)}^{2}}}\\ -arctg\frac{\left(x_{0}+a)(z_{0}-c\right)}{(y_{0}-b)\sqrt{{\left(x_{0}+a\right)}^{2}+{\left(y_{0}-b\right)}^{2}+{\left(z_{0}-c\right)}^{2}}}] \end{array}$$

Note that contribution due to the **L** surface is derived from this same formula but with opposite, “*b*” sign. Whereas, due to the surface **S** and **N**, the *x* and *y* variable as well as *a* and *b* constant must be changed in places. Only the *B_x_* component is affected by these surfaces without any contribution into the *B_y_*. The induction therefore, due to the whole solenoid and/or more solenoids is, found by application of the respective superposition procedure.

Similarly, a cylindrical magnet of radius *R* and length 2*c* (Figure 4b) magnetized along the *z*-axis can be substituted by an equivalent cylindrical solenoid with current flowing along its cylindrical surface. In this case, for the point *P* (*x*_0_, *y*_0_, *z*_0_) located on the *x*_0*z*_ surface after double integration of Equation (3) one obtains:

(5) $$B_{x}=\frac{\mu RFz_{0}}{\pi }\int_{0}^{\pi }\frac{\cos\phi d\phi }{\sqrt{T_{1}T_{2}}(\sqrt{T_{1}}+\sqrt{T_{2}})}\;B_{y}=0\;B_{z}=\frac{\mu RF}{4\pi c}\int_{0}^{\pi }\left(\frac{z_{0}-c}{\sqrt{T_{2}}}-\frac{z_{0}+c}{\sqrt{T_{1}}}\right)\frac{(R-x_{0}\cos\phi )d\phi }{T_{3}}$$

where:

(6) $$T_{1}=R^{2}+x_{0}^{2}+{\left(c+z_{0}\right)}^{2}-2x_{0}R\cos\phi$$

(7) $$T_{2}=R^{2}+x_{0}^{2}+{\left(c-z_{0}\right)}^{2}-2x_{0}R\cos\phi$$

(8) $$T_{3}=R^{2}+x_{0}^{2}-2x_{0}R\cos\phi$$

The elliptic integral in Equation (5), can be solved numerically using the appropriate algorithm for small time intervals.

## 3. Experimental Results and Discussion

**Figure 5:**
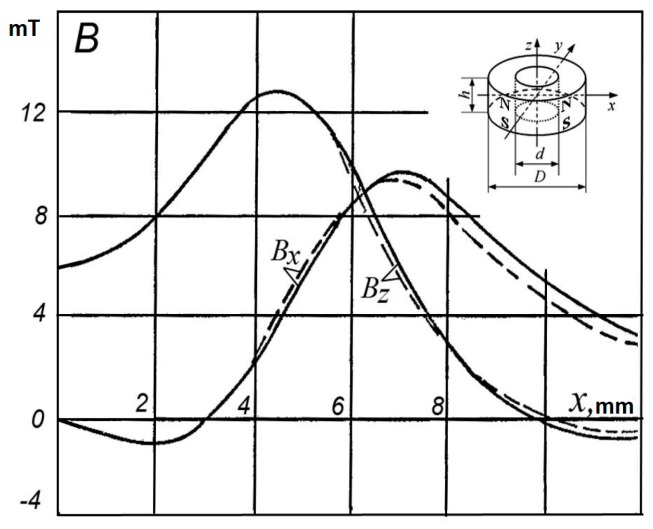
Distribution of *B_x_* and *B_z_* components along the *x*-axis due to axially magnetized ring-shaped magnet (made of a material 1BI of dimensions: *D* = 11.5 mm, *d* = 7.5 mm, *h* = 4.4 mm) for selected point at *y* = 0 and *z* = 5.2 mm (solid lines-experimental data, dashed lines-calculated).

Comparison of the analysis and measurement results of distribution of the vector components *B_x_* and *B_z_* of the magnetic flux density along the *x*- and *z*-axis for the axially magnetized ring-shaped permanent magnet made of a material type 1BI of dimensions as follows: *D* = 11.5 mm, *d* = 7.5 mm, *h* = 4.4 mm (for selected point at *y* = 0 and *z* = 5.2 mm) is shown, for example, in Figure 5. 

Similar measurements and comparative calculations were made to verify the value of the force acting on the steel ball in the magnetic field produced by a bar magnet made of 24BA210 material with dimensions of 11.5 mm × 7.0 mm × 11.5 mm. To estimate the resistance of the ball sensor to the external vibrations and mechanical shocks, the ball diameter was changed from 0.5 mm to 1.6 mm, referring the force value to the weight of the ball. A sufficient consistency (±5%) with the measured results on the physical switch model has been found [17].

As results from Equation (1) indicate, the direction of the force *P_x_* (affecting the ball) is in line with the increase in the induction value. It is zero wherever the flux density is equal zero or indicates extreme value. However, at points of its maximum the ball is kept in stable steady state whereas, at the minimum (including zero-singular points) it becomes unstable (under the magnet movement along the *x*-axis). Therefore, to increase the force *P_x_* value one has to increase either the value or gradient of the magnetic flux density. However, the increase in the induction value is limited either due to a magnetic saturation of the flux conducting elements or limited by restriction of a magnetic energy provided by the permanent magnets. In turn, a high gradient can be achieved by suitable design of the magnetic circuit structure. Such circuit should ensure suitable distribution of the magnetic induction along the trajectory of motion of the ball with alternately changing values of minima and maxima. It may, for example, be in the form of a ring axially magnetized permanent magnet (as in Figure 2). However, for such axially symmetrical system the component *B_x_* along the *x*-axis is equal to resultant *B*. If therefore permanent magnet 3 will be placed so that the ball moves along the *x*-axis, and the center of the ball is found between the singular point *K* and one of the points of the maximum induction value (points *0* or *L*)—for example at the point *S*—therefore, the electromagnetic force will be directed to the point *L*. It results in snap action of the ball moving to the right and closing the respective contacts. If, however, the magnet is moved so that a singular point *K* appears on the right side of the ball center thus the electromagnetic force is directed oppositely—to the point *0*. In this case, the ball moves in a step way to the left and closes the second pair of contacts respectively. Application of the two ring-shaped permanent magnets magnetized axially but opposite located [3], allows to increase respectively the induction gradient at the area of the ball location. This allows to get a controllable switch performance with a snap-action without the need for a return spring (return force is due to the magnetic repulsion force) [2,3,16].

**Figure 6:**
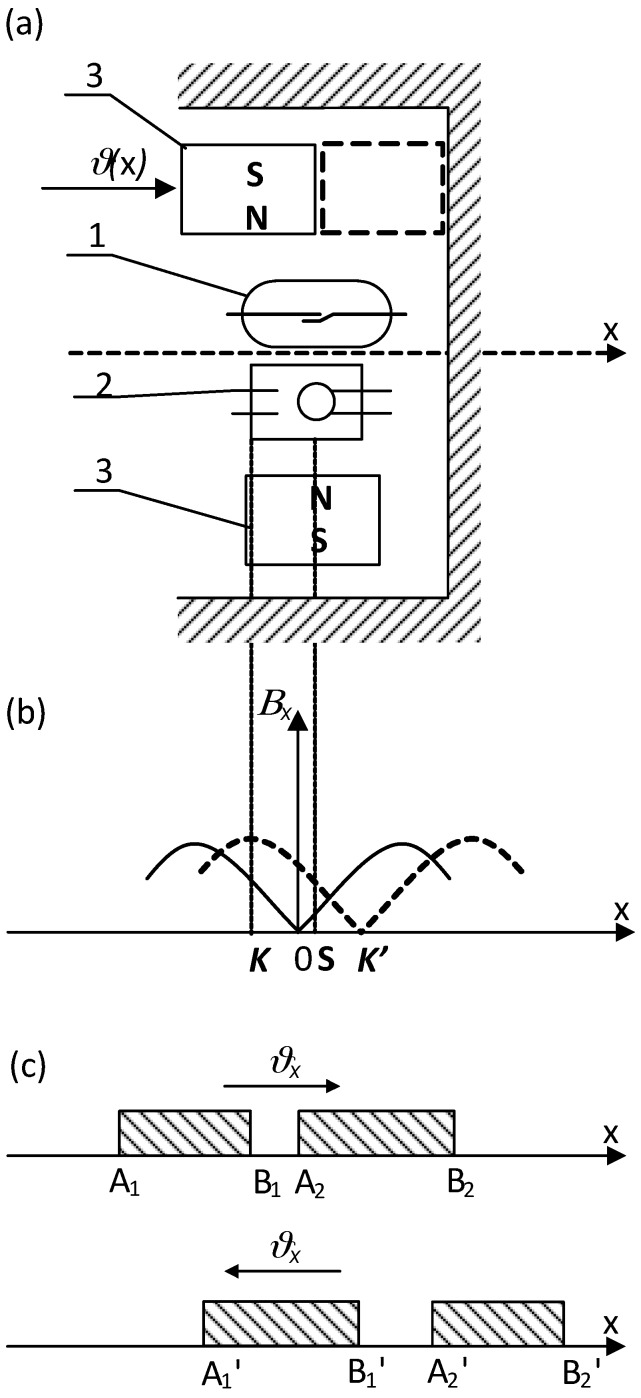
The reed (1) and/or the magnetic ball contact sensor (2) controlled by means of two rod permanent magnets (3) (**a**); (**b**)—distribution of a magnetic flux density *B_x_* (*K*, *K*’—singular points); (**c**)—areas of the reed switch operation A_1_…A_2_, B_1_…B_2_—contact closure and release respectively.

The required distribution of the magnetic flux value can also be obtained by application of the planar permanent magnets. For a system made of two such magnets 3 arranged as shown in Figure 6a, respective distribution of the magnetic induction with alternating maxima and minima occurs along the *x*-axis.

The displacement of the singular point from the *K* to *K*’ position is obtained by moving the upper permanent magnet on the *x*-axis (shown by dotted lines). Under an influence of the electromagnetic force directed to the point of maximum value of the flux density, ball 2 moves to the left. The mutual interaction of the permanent magnets not only provides the snap action of the ball contact but also results in sudden displacement of the moving magnet and its placement into extreme positions.

**Figure 7:**
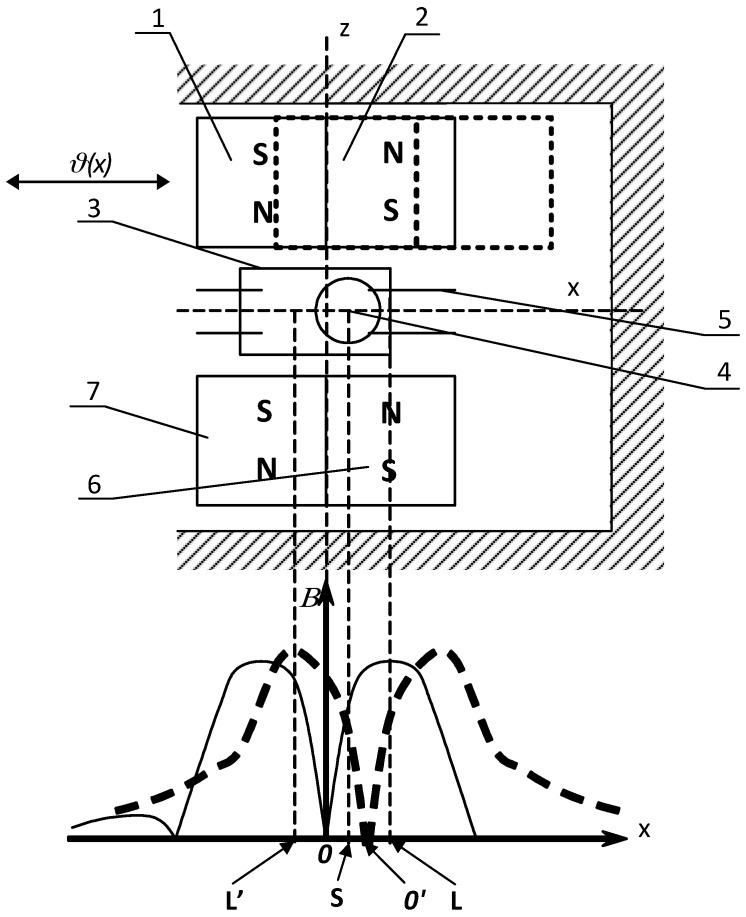
Magnetic contact sensor controlled by means of two rod-shaped permanent magnets (field curves marked with solid and dashed line correspond to respective position of the driving magnet).

Figure 7 shows a system in which, thanks to the unique composition of the permanent magnet materials, a very high gradient is produced within the area of the ball location. It significantly increases the value of the electromagnetic force. 

Applied permanent magnets (1), (2), (6) and (7) are placed on two opposite sides of the ball contact along the *x*-axis. Wherein, each of the pairs of magnetic poles is located contrary to each other, and relative to the opposite pair. A pair of magnets (6) and (7) is immovable, whereas magnets (1) and (2) move on the *x*-axis on either side of the transverse *z*-axis. At the starting position, these two pair of magnets are arranged to be located symmetrically with respect to *z*-axis. The minimum (zero) value of the magnetic induction (singular point) occurs at the point *0*. Therefore, ball (4) cannot be kept at this point of unstable equilibrium and moves to one of the terminal positions; for example, to the right to a point L of a maximum induction closing as a result the contact (5). When move the pair of the magnets (1–2) to the right (Figure 7—dotted line), the singular point *0’* will be shifted to the right of the center of the ball (4) (point S). As a result, the electromagnetic force will be directed to point L’ with a maximum induction. The ball (4) moves therefore, to the left and changes over the contact. After release the moving pair of magnets (1–2) it returns immediately to the symmetrical position (multi-function device of a bistable operation). Note, however, that the length of movement of pair of magnets (1–2) should be smaller than the dimension of one magnet on the *x*-axis. Otherwise, automatic return to the in-put magnets’ position will never occur.

## 4. Conclusions

Highly miniaturized and integrated structures of switches with magnetically driven contacts should meet requirements, if about a high reliability and a high speed of operation as well as respective value of the contact force. This requires the use of integrated permanent magnets with significant magnetic induction values and significant gradients of its change along the small distance of the magnet movement. To fulfill these requirements, a respective knowledge on the magnetic field distribution with the appropriate spatial location of the singular points of the field is needed. The ball switch can be successfully used both as a changeover relay and a detector of the singular points under designing. The magnetic field distribution can be successfully obtained using the method of an equivalent solenoid. Most authors determine the distribution of the magnetic field using the finite element method FEM [18]. The correctness of such reasoning and behavior has been proved by results of calculations and measurements with practically sufficient accuracy (±5%). 

It is worth emphasizing that the miniature reed switches provide a continuous contact current of about a few milliamps and an electrical strength of not less than 10 Vdc [1,2]. However, applying them to given load conditions (dry circuit, low level load etc.) requires, first of all, proper selection of the contact material. This also applies to the ball connector in which the use of a mercury wetting (if possible) effectively prevents sticking of the contact. In all cases, however, the maximum speed of the contact movement should be as high as possible (snap action). It should also be remembered that the movement length of a pair of magnets is relatively small and does not usually exceed half the length of the magnet used (Figure 7). In any case, however, it is not recommended to use these switches for breaking any inductive load.
